# Psychosocial impacts of the COVID-19 pandemic from a cross-sectional Survey of people living with HIV in Washington, DC

**DOI:** 10.1186/s12981-023-00517-z

**Published:** 2023-05-09

**Authors:** Anne K. Monroe, Paige E. Kulie, Morgan E. Byrne, Brittany C. Wilbourn, Shannon K. Barth, Jenna B. Resnik, David M. Huebner, Michael A. Horberg, Amanda D. Castel, Alan E. Greenberg, the DC Cohort Executive Committee

**Affiliations:** 1 Department of Epidemiology, Milken Institute School of Public Health, George Washington University, 950 New Hampshire Avenue, Washington, DC, NW 20052 USA; 2 Department of Prevention and Community Health, Milken Institute School of Public Health, George Washington University, Washington, DC, USA; 3 Kaiser Permanente Mid Atlantic States, Rockville, MD, USA

**Keywords:** COVID-19, HIV, Mental health, Psychosocial impact

## Abstract

**Background:**

COVID-19 has not only taken a staggering toll in terms of cases and lives lost, but also in its psychosocial effects. We assessed the psychosocial impacts of the COVID-19 pandemic in a large cohort of people with HIV (PWH) in Washington DC and evaluated the association of various demographic and clinical characteristics with psychosocial impacts.

**Methods:**

From October 2020 to December 2021, DC Cohort participants were invited to complete a survey capturing psychosocial outcomes influenced by the COVID-19 pandemic. Some demographic variables were also collected in the survey, and survey results were matched to additional demographic data and laboratory data from the DC Cohort database. Data analyses included descriptive statistics and multivariable logistic regression models to evaluate the association between demographic and clinical characteristics and psychosocial impacts, assessed individually and in overarching categories (financial/employment, mental health, decreased social connection, and substance use).

**Results:**

Of 891 participants, the median age was 46 years old, 65% were male, and 76% were of non-Hispanic Black race/ethnicity. The most commonly reported psychosocial impact categories were mental health (78% of sample) and financial/employment (56% of sample). In our sample, older age was protective against all adverse psychosocial impacts. Additionally, those who were more educated reported fewer financial impacts but more mental health impacts, decreased social connection, and increased substance use. Males reported increased substance use compared with females.

**Conclusions:**

The COVID-19 pandemic has had substantial psychosocial impacts on PWH, and resiliency may have helped shield older adults from some of these effects. As the pandemic continues, measures to aid groups vulnerable to these psychosocial impacts are critical to help ensure continued success towards healthy living with HIV.

**Supplementary Information:**

The online version contains supplementary material available at 10.1186/s12981-023-00517-z.

## Introduction

The toll in terms of lives lost from the Coronavirus disease 2019 (COVID-19) pandemic has been staggering, with close to 85 million reported cases and over 1 million deaths as of June 2022 in the United States (U.S.), and over 6.2 million deaths worldwide [1]. However, the psychosocial impacts of the pandemic are even wider-reaching than case and mortality counts indicate.

The psychological impacts (e.g., fear, stress, anxiety, depression) of COVID-19 [2–7] and efforts to limit the spread of COVID-19 (e.g., social distancing, quarantine, isolation, stay-at-home orders) [8] have resulted in widespread social disruption (e.g., school closure, unemployment, travel restriction, limited or virtual health care services) which may have the unintended consequence of exacerbating the psychological impact [9–11]. Psychiatric disorders and social challenges are also highly prevalent among people with HIV (PWH) [12, 13] and may be intensified by COVID-19.

Early evidence suggests widespread psychosocial impacts of the COVID-19 pandemic among PWH [14, 15]: income reduction [16, 17], disruptions in substance use treatment [18, 19], increased loneliness [20], particularly among women [21], and increased anxiety and depression symptoms [22–24].

The District of Columbia (DC) has been a COVID-19 hotspot at various points in the pandemic [25–27] and is an established HIV hotspot [28]. In this setting, we assessed the psychosocial impacts of the COVID-19 pandemic and its containment measures in a large cohort of patients receiving HIV care in Washington DC. The overall framework for this research was the socio-ecological model [29]. This model considers the factors that are most likely to influence HIV-related behavior and outcomes, many of which may have been influenced by the pandemic. This analysis examined demographic and clinical characteristics associated with psychosocial impacts of the COVID-19 pandemic among PWH in Washington, DC. Our goal with this work was to explore who in DC was most vulnerable to psychosocial impacts due to the pandemic. We hypothesized that individuals who in older age groups would be most vulnerable to adverse psychosocial impacts of the pandemic. This analysis was primarily descriptive and lays the groundwork for future longitudinal analyses of HIV-related outcomes during the pandemic. This may ultimately help us tailor interventions to support people with HIV to maintain undetectable HIV RNA and to avoid adverse HIV-associated outcomes.

## Methods

### Setting and participants

The DC Cohort is a longitudinal observational cohort study of HIV-infected persons receiving care at 14 clinical sites in DC [28]. With over 11,000 enrolled participants, it is the largest city-wide cohort of PWH in the U.S. and is representative of PWH in DC, with the majority of participants being male and Black [28]. Patient-level data are routinely collected from electronic health records (EHRs) on socio-demographics, clinic visits, and clinical factors including laboratory values and prescribing information. Data are imported into a centralized database and processed into analytic files via SAS.

### Recruitment and screening

Active DC Cohort participants aged 18 and older were approached in person or by telephone, email, or during a telehealth visit and provided additional information about a COVID-19 survey. A survey link was sent to interested participants via email or text. Participants were remunerated with a $25 gift card for their participation.

### Survey

The COVID-19 survey is an ongoing a cross-sectional survey, with data collection starting October 30, 2020. Data were collected and managed using Research Electronic Data Capture (REDCap), a secure, web-based software platform. The survey was available in both English and Spanish. Participants who did not have access to a computer or smartphone were administered the survey by phone. Informed consent was embedded in the survey which was approved by the George Washington University IRB. The survey included the following domains, adapted from existing instruments [30–38]: socio-demographics, healthcare access, pre-existing medical conditions, household contacts, COVID-19 symptoms and testing, impact of COVID-19, risk perceptions related to COVID-19, depressive symptoms, post-traumatic stress disorder, anxiety, insomnia, tobacco product use, sexual risk behaviors, COVID-19 stigma, ART adherence, and telehealth. Validated instruments were used whenever available [31, 33, 38]. Additional file 1: Appendix 1 contains the survey instrument.

### Variables of interest

Four domains of psychosocial impacts of COVID-19 were assessed:

1. Financial/employment: household income decreased; lost health insurance; negative impact on paying rent/mortgage; negative impact on getting food; lost housing.
2. Mental health: feeling anxious increased; quality of life decreased; quality of sleep decreased; response of “Yes” to anhedonia and/or depressed mood question on PHQ-2 screening [38].
3. Decreased social connection: feeling connected to family decreased; feeling connected to friends decreased.
4. Substance use: alcohol use increased; illicit drug use increased.

Participants indicated the degree to which they experienced each psychosocial impact using a four-point scale (highly increased, increased, decreased, highly decreased). Responses were collapsed such that “increased” and “highly increased” were combined to “increased” while responses of “decreased” and “highly decreased” were combined to “decreased.” The psychosocial impacts were first examined individually (13 responses in the 4 domains above). Subsequently, they were examined by domain. If a respondent selected “yes” to at least one response in a given domain, that domain was considered impacted.

Demographic and clinical characteristics from the COVID survey included: age (categorical: 16–38 years; 39–49 years; 50 + years), gender identity (male; female), race/ethnicity (Non-Hispanic Black; Non-Hispanic white; Hispanic, any race; other), education (at least some high school; at least some college), self-reported underlying medical conditions (0 or ≥ 1), and year of survey completion. All participants completed the survey once. Some completed it in the calendar year 2020 and others completed it in the calendar year 2021.

Additional demographic and clinical characteristics included the following from the DC Cohort database: HIV mode of transmission (men who have sex with men (MSM), high risk heterosexual (HRH), IDU, or a composite group “Other” consisting of none, missing, or unknown), duration of HIV infection, and most recent measure (between 1/12020 and 12/31/2021) of CD4 cell count (cells/µl) and HIV RNA suppression (viral suppression (VS)).

### Statistical analysis

Analyses for this study included survey data through December 31, 2021 linked to DC Cohort medical record data. Statistical analyses included descriptive statistics. Results are shown as frequencies (%) for categorical variables and median (interquartile range (IQR)) for variables.

Multivariable logistic regressions to evaluate the association between demographic and clinical characteristics and the psychosocial impacts were performed for the financial/employment, mental health, decreased social connection, and substance use variables individually and as overarching domains.

Adjusted Odds Ratios (aOR) and 95% Confidence Intervals (95% CI) were estimated by adjusting for age, gender, HIV transmission risk, race/ethnicity, education, self-reported underlying conditions, VS, CD4, and survey year. p < 0.05 was considered statistically significant.

An additional multivariable logistic regression model for psychosocial impact category was fit excluding CD4 and VS status. We performed the additional modeling because not all survey respondents had a CD4 count and/or HIV RNA and we wanted to evaluate the demographic and clinical characteristic associations in the overall sample, regardless of whether lab values were present. Analyses were conducted using SAS, version 9.4 (Cary, NC, USA).

## Results

During the survey period, 891 participants completed the survey. The response rate was 41%. Of those, 748 had a CD4 value available and 751 had an HIV RNA lab value available (last measured value after 1/1/20 through 12/31/21). As shown in Table 1, the median age of the sample was 46 years old. Most participants (75.7%) were of non-Hispanic Black race/ethnicity. Over half (55.6%) were employed full-or part-time as of 1/1/20. Most of the sample for whom HIV RNA results were available was virally suppressed (717/748, or 95.9%).

**Table 1:** Demographic and clinical characteristics of DC Cohort COVID-19 Survey participants, N = 891, 2020–2021

Characteristic	Total (N = 891)^a^
	N (%)
Age (median, IQR) in years (n = 891)	46.0 (37.0–54.0)
Age tertile category (n = 891)	
16–40 Years	296 (33.2)
41–51 Years	296 (33.2)
52 + Years	299 (33.6)
Gender identity (n = 881)	
Male	576 (65.4)
Female	305 (34.6)
Race/ethnicity (n = 870)	
Non-Hispanic Black	659 (75.7)
Non-Hispanic White	115 (13.2)
Hispanic, any race	50 (5.7)
Other (American Indian/Alaska native, Asian, Native Hawaiian/Pacific Islander, Bi/multiracial)	46 (5.3)
Education (n = 878)	
At least some high school	339 (38.6)
At least some college	539 (61.4)
Pre-pandemic work status (as of 1/1/20) (n = 891)	
Employed full- or part-time	495 (55.6)
Unemployed	133 (14.9)
Other^b^	263 (29.5)
Household composition (n = 887)	
Lives alone	381 (43.0)
Lives with others	506 (57.0)
Relationship status (n = 891)	
Divorced, separated, or widowed	154 (17.3)
Married or live-in partner	203 (22.8)
Single, Other, Decline to Answer	534 (59.9)
Housing status (n = 891)	
Rental	530 (59.5)
Own	209 (23.5)
Homeless	7 (0.8)
Other^c^	145 (16.3)
Location of residence (n = 884)	
DC	680 (76.9)
MD	154 (17.4)
VA	43 (4.9)
Other state	7 (0.8)
Presence of the following comorbid conditions	
Angina or coronary heart disease	27 (3.0)
Asthma	163 (18.3)
Cancer	86 (9.7)
Chronic lung disease	63 (7.1)
Depression	313 (35.1)
Myocardial infarction	21 (2.4)
High blood pressure	374 (42.0)
Kidney disease	62 (7.0)
Overweight or obesity	194 (21.8)
Stroke	37 (4.2)
Type II diabetes	110 (12.3)
Decline to answer	11 (1.2)
Self-reported underlying medical conditions in addition to HIV* (n = 891)	
0	179 (20.1)
≥ 1	712 (79.9)
Survey year	
2020	135 (15.2)
2021	756 (94.8)
Median duration of HIV infection (yrs) (IQR) (n = 884)	16.0 (10.0, 24.0)
CD4 ≥ 200 cells/ml^d^ (n = 748)	717 (95.9)
HIV RNA < 200 copies/ml^d^ (n = 751)	674 (89.7)
HIV transmission risk factor^e^ (n = 891)	
MSM	398 (44.7)
HRH	240 (26.9)
IDU	39 (4.4)
Other^e^	214 (24.0)

^a^Totals may not sum to N due to missing data, participants had an available COVID survey before 12/31/2021 and are enrolled in the DC Cohort

^b^Other includes student, homemaker, retired, disabled

^c^Other includes lives with parent/friends, lives in rooming/halfway/group home, lives in residential drug facility, lives in assisted living, other

^d^Last lab value after 1/1/2020 up to dataset closure on 12/31/2021: 4 sites not submitting HIV RNA labs considered missing

^e^MSM includes MSM/IDU; Other includes Hemophilia, blood transfusion, Perinatal, other, unknown

*Self-reported presence of any comorbid condition listed in the survey (“ > 1”) OR the response “I have not been told I have any of the above conditions” (“0”) to the question “To your knowledge have you ever had any of the following medical conditions?”

**Fig. 1:**
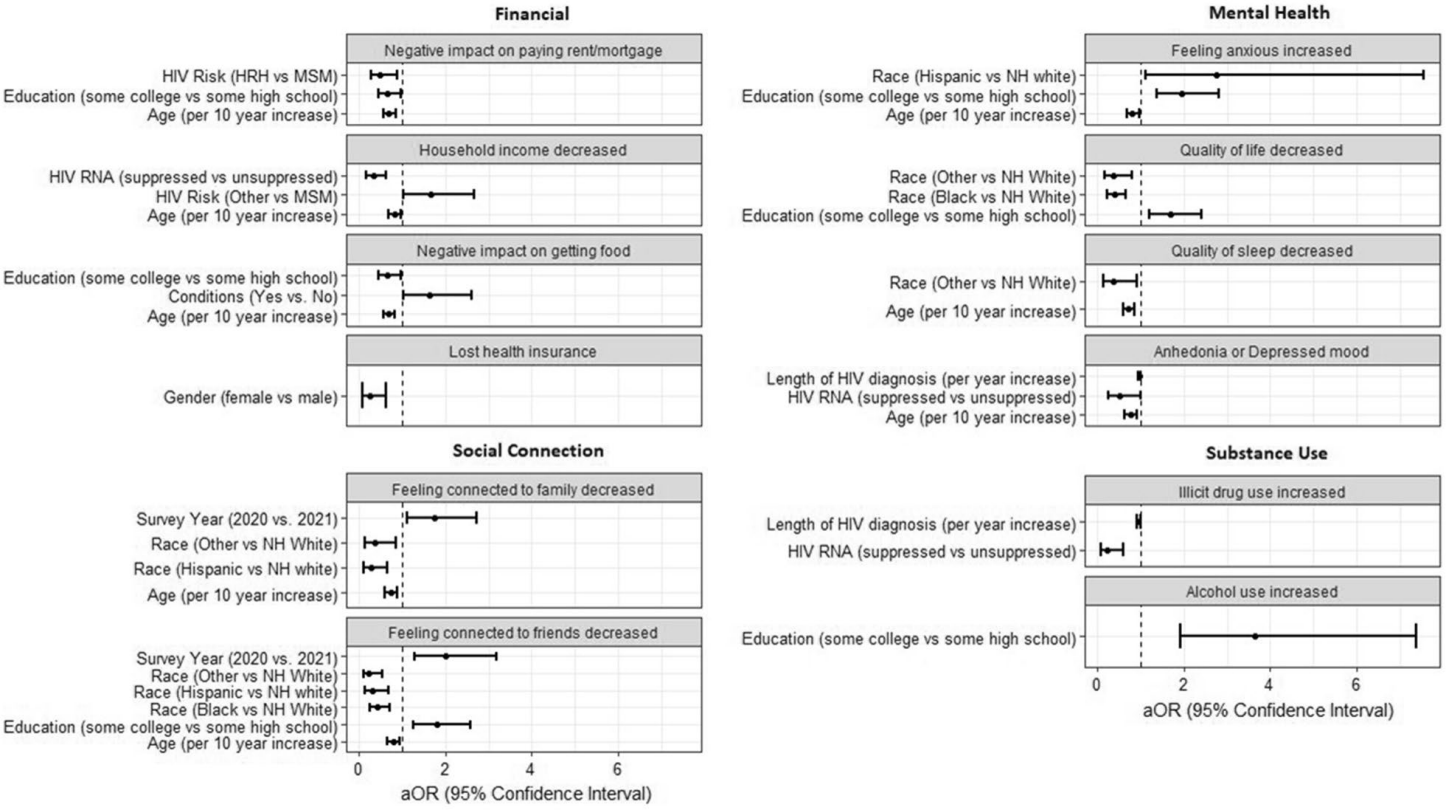
Factors associated with individual psychosocial impacts, DC Cohort 2020–2021

The results for each psychosocial impact category will be discussed in the text, which reference relevant information from the following sources: Tables 2, 3; Fig. 1.

**Table 2:** Prevalence of psychosocial impacts, individual and by category, DC Cohort 2020–2021

Impact	Total
	N (%)
Financial/employment	495 (55.7)
Negative impact on paying rent/mortgage	258 (29.0)
Household income decreased	306 (34.3)
Negative impact on getting food	240 (26.9)
Lost health insurance	51 (5.8)
Lost housing	45 (5.1)
Mental health	692 (77.7)
Feeling anxious increased	445 (49.9)
Quality of life decreased	415 (46.6)
Quality of sleep decreased	291 (32.7)
Anhedonia or depressed mood (PHQ-2 results)	317 (35.6)
Social connection	460 (51.6)
Feeling connected to family decreased	345 (38.7)
Feeling connected to friends decreased	429 (48.1)
Substance use	146 (17.9)
Alcohol use increased	121 (15.1)
Illicit drug use increased	63 (8.2)

**Table 3:** Factors associated with psychosocial impact categories, DC Cohort 2020–2021

	Financial/employment^a^ (n = 690)		Mental health^b^ (n = 690)		Decreased social connection^c^ (n = 690)		Increased substance use^d^ (n = 626)	
	aOR (95%CI)	P-value	aOR (95%CI)	P-value	aOR (95%CI)	P-value	aOR (95%CI)	P-value
Age (continuous), per 10 year increase	0.76 (0.63–0.90)	0.0022	0.72 (0.58–0.89)	0.0031	0.77 (0.64–0.91)	0.0030	0.72 (0.56–0.92)	0.0094
Gender identity								
Male	Ref.		Ref.		Ref.		Ref.	
Female	0.83 (0.53–1.31)	0.4302	1.03 (0.61–1.74)	0.9208	1.04 (0.66–1.62)	0.8765	0.40 (0.19–0.83)	0.0144
Race/ethnicity								
Non-Hispanic White	Ref.		Ref.		Ref.		Ref.	
Non-Hispanic Black	1.43 (0.88–2.33)	0.1532	0.64 (0.32–1.19)	0.1723	0.42 (0.25–0.70)	0.0010	0.62 (0.34–1.13)	0.1145
Hispanic, any race	1.43 0.66–3.18)	0.3684	6.00 (1.12–111.33)	0.0907	0.26 (0.12–0.58)	0.0011	0.64 (0.23–1.67)	0.3722
Other (American Indian/Alaska native, Asian, Native Hawaiian/Pacific Islander, Bi/multiracial)	1.11 (0.49–2.50)	0.8083	0.62 (0.22–1.84)	0.3669	0.25 (0.11–0.58)	0.0011	0.56 (0.19–1.52)	0.2725
Education								
At least some high school	Ref.		Ref.		Ref.		Ref.	
At least some college	0.59 (0.41–0.85)	0.0043	1.82 (1.19–2.81)	0.0061	1.54 (1.08–2.19)	0.0167	3.31 (1.87–6.15)	< 0.0001
Self-reported underlying medical conditions								
0	Ref.		Ref.		Ref.		Ref.	
≥ 1	1.10 (0.73–1.64)	0.6595	1.56 (0.96–2.52)	0.0713	1.32 (0.88–1.97)	0.1775	0.98 (0.58–1.67)	0.9280
Survey year								
2021	Ref.		Ref.		Ref.		Ref.	
2020	1.34 (0.85–2.14)	0.2141	1.60 (0.91–2.97)	0.1159	1.84 (1.17–2.93)	0.0095	1.23 (0.64–2.26)	0.5209
Length of diagnosis (per year)	1.00 (0.98–1.02)	0.7387	1.00 (0.98–1.02)	0.9133	1.00 (0.98–1.02)	0.8933	0.99 (0.96–1.02)	0.4569
AIDS CD4 ≥ 200 cells/ml vs. < 200 (Ref.)	1.29 (0.50–3.26)	0.5911	0.41 (0.09–1.39)	0.1915	0.52 (0.21–1.25)	0.1495	0.86 (0.29–2.82)	0.7856
Suppressed HIV RNA < 200 copies/ml vs > 200 (Ref.)	0.48 (0.23–0.97)	0.0476	0.80 (0.30–1.89)	0.6308	1.50 (0.78–2.91)	0.2242	0.48 (0.22–1.06)	0.0616
HIV mode of transmission								
MSM	Ref.		Ref.		Ref.		Ref.	
Hetero	0.93 (0.55–1.56)	0.7702	0.90 (0.49–1.67)	0.7397	0.85 (0.50–1.42)	0.5218	2.03 (0.94–4.33)	0.0688
IDU	1.27 (0.58–2.83)	0.5446	1.45 (0.59–3.86)	0.4332	1.02 (0.47–2.22)	0.9594	2.04 (0.53–6.54)	0.2571
Other	1.60 (1.00–2.57)	0.0493	1.14 (0.65–2.03)	0.6624	0.92 (0.58–1.45)	0.7150	0.80 (0.39–1.54)	0.5183

^a^Reported at least 1 of the following: Household income decreased, Lost health insurance, Lost housing, Negative impact on paying rent/mortgage, Negative impact on getting food

^b^Reported at least 1 of the following: Quality of life decreased, Feeling anxious increased, Quality of sleep decreased, PHQ-2 positive (both questions answered affirmatively)

^c^Reported at least 1 of the following: Feeling connected to family decreased, Feeling connected to friends decreased

^d^Reported at least 1 of the following: Use of illicit drugs increased; Use of alcohol increased

### Psychosocial impact category: financial/employment

As shown in Table 2, the overall prevalence of negative financial/employment impact was 55.7%, with a decrease in household income being reported as the most prevalent individual response (34.3%).

Figure 1 illustrates factors associated with individual responses within the financial/employment impact domain. Figure 1 shows that females were less likely to lose their health insurance than males. In addition, Fig. 1 shows that HRH were more likely to report a negative impact on paying rent and/or mortgage than MSM, and those with suppressed HIV RNA were less likely to report losing their housing compared to those with unsuppressed HIV RNA.

Table 3 examines the overall financial/employment domain, and demonstrates that older age was inversely associated with an adverse financial/employment impact (aOR per 10 year age increase 0.76, 95% CI 0.63, 0.90), p = 0.0022). In addition, compared to those who had completed at least some high school, those who had completed at least some college were less likely to report negative financial/employment impact (aOR 0.58, 95% CI 0.41, 0.84, p = 0.0037). Finally, individuals who were virally suppressed were less likely to report a negative financial/employment impact (aOR 0.48, 95% CI 0.23, 1.00, p = 0.0490).

### Psychosocial impact category: mental health

Table 2 shows that 77.7% of respondents overall reported a negative mental health impact. Feeling anxious (49.9%) and reporting decreased quality of life (46.6%) were most common.

Comparisons in Fig. 1 demonstrate that Hispanic individuals were more likely to report increased anxiety compared to non-Hispanic white individuals. Also, individuals with suppressed HIV RNA were less likely to report feelings of anhedonia/depression than individuals with unsuppressed HIV RNA.

Table 3 demonstrates that older age was inversely associated with an adverse mental health impact (aOR per 10 year age increase 0.72, 95% CI 0.58, 0.89), p = 0.0031).

Additionally, compared to those who had completed at least some high school, those who had completed at least some college were more likely to report a negative mental health impact (aOR 1.74, 95% CI 1.13, 2.67, p = 0.0121). Finally, compared to non-Hispanic White individuals, non-Hispanic Black individuals were less likely to report a negative mental health impact (aOR 0.66, 95% CI 0.35, 1.25, p = 0.1998), though this finding only achieved statistical significance in multivariable models which did not include lab values, as there were more participants included in those models (data not shown).

### Psychosocial impact category: decreased social connection

The overall prevalence of reporting decreased social connection was 51.7% (Table 2). A decrease in connections to friends (48.1%) was reported more frequently than a decrease in connections to family (38.7%).

Table 3 demonstrates that older age was inversely associated with reporting decreased social connection (aOR per 10 year age increase 0.77, 95% CI 0.64, 0.91), p = 0.0030) Additionally, compared to those who had completed at least some high school, those who had completed at least some college were more likely to report decreased social connection (aOR 1.48, 95% CI 1.04, 2.11, p = 0.0285). Compared to non-Hispanic White individuals, non-Hispanic Black, Hispanic, and other races were less likely to report decreased social connection (p-values p = 0.0014, p = 0.0016, and p = 0.0015, respectively). Lastly, comparing results of the one-time survey that were completed in 2021, survey respondants in 2020 were more likely to report decreased social connection (aOR 1.80, 95% CI 1.14, 2.85, p = 0.0120).

### Psychosocial impact category: substance use

Table 2 highlights the 17.9% overall prevalence of adverse substance use impact. An increase in alcohol use (15.1%) was more commonly reported than increased illicit drug use (8.2%).

Figure 1 demonstrates that individuals with suppressed HIV RNA were less likely to report increased substance use.

Table 3 demonstrates that older age was inversely associated with reporting increased substance use (aOR per 10 year age increase 0.72, 95% CI 0.56, 0.92), p = 0.0094) CCompared to those who had completed at least some high school, those who had completed at least some college were more likely to report increased substance use (aOR 3.29, 95% CI 1.82, 5.97, p < 0.0001). Finally, compared to males, females were less likely to report increased substance use (aOR 0.41, 95% CI 0.20, 0.86, p = 0.0182).

## Discussion

In this city-wide longitudinal cohort, many PWH reported psychosocial impacts of the COVID-19 pandemic, including increased anxiety, decreased quality of life, financial difficulties, and feelings of isolation. As the pandemic continues and some emergency supports that were established early in the pandemic are removed, vulnerable individuals who are already at risk for worse HIV outcomes may suffer more. Considering the socio-ecological model [29], factors at all levels (individual, interpersonal, community, institutional and structural) can influence health outcomes. The factors examined in this study were primary individual and interpersonal; the DC Cohort also has ongoing investigations of institutional and health system level responses to the pandemic that may have influenced HIV outcomes. To continue to advance the goals of the Ending the HIV epidemic, i.e., to keep people with HIV on therapy so that they maintain viral suppression and do not transmit the virus to others, understanding and addressing these factors is key.

Adverse psychosocial impacts of the pandemic among PWH have been previously reported [14]. In our sample, a large number of respondents (27%) reported difficulty getting food, and even more (29%) reported difficulty paying rent/mortgage. Even pre-pandemic, housing costs in the DC metropolitan area were rising at a faster pace than incomes [39], and unstable housing or homelessness was associated with worse HIV outcomes [40–42].

Multiple studies have demonstrated increased stress and mental health symptoms among PWH resulting from the COVID-19 pandemic [15, 43]. Many of our respondents reported increased feelings of anxiety and decreased sleep quality. Over one-third (36%) of our participants reported one or two of the hallmark symptoms of depression, anhedonia, or depressed mood. This is similar to other research groups’ findings during the pandemic. In a multinational sample, 28% of people with HIV had a positive screen for depression [22]. A large survey of MSM in the US, 10% of whom had HIV, showed that 72% had increased anxiety as a result of the pandemic [44]. The escalation of anxiety due to the pandemic has many possible explanations: concern regarding contracting COVID-19 and the potentially increased severity of COVID-19 among PWH, finances, and access to care, among others [15].

The AGE_h_IV Cohort in the Netherlands, which includes participants aged 45 and older at time of enrollment (2010–2012), also examined the impact of social distancing as a result of the pandemic and health-related quality of life, depressive symptoms [45]. They described additional psychosocial impacts as well. The investigators found that PWH were more likely to report increased alcohol use. About 8.5% reported clinically significant depressive symptoms, with no difference by HIV status. Concerns about getting ill with COVID were inversely associated with self-reported health-related QOL and associated with depressive symptoms.

Another major impact of the pandemic was on social isolation and loneliness. Individuals with HIV are already at increased risk of loneliness due to the stigma surrounding HIV [46], and our results, as well as others, have demonstrated increased social isolation, with respondents noting feeling disconnected from family and friends [20].

Looking across the domains examined, older age was protective against adverse outcomes in all four domains. Prior research has shown similar findings [47–51], and those findings have attributed to increased resilience in older adults that allows them to weather stressors better. Younger people with HIV often fare worse in terms of HIV care continuum outcomes [52], stemming from less stability in their social situations. Given the domains of interest in this analysis: financial/employment, mental health, decreased social connection, and increased substance use, older age is likely protective against these impacts due to having more life experience, being better able to navigate systems to maintain benefits and/or connect with needed resources, and less susceptibility to substance use.

Education had various effects on the psychosocial impacts. Individuals with more education were less likely to have financial/employment impacts, possibly reflecting that these individuals were able to continue their employment working at home. Fields that were particularly impacted by the pandemic included hospitality and food service industry jobs [53], which are typically filled by individuals with lower education level. The opposite effect of education on other impacts was demonstrated, i.e., individuals with higher education level were more likely to have mental health, social connection, or increased substance use impacts. The cause of these findings is likely multifactorial. Assuming that individuals with higher education levels are more likely to have jobs that allow them to work from home, increased mental health issues may have been related to working from home and balancing other responsibilities simultaneously. Alternatively, individuals with higher education may be employed in a health care setting, which was a particularly stressful environment during the pandemic. Decreased social connection among individuals with higher education may result from having the resources to live alone, be working alone at home, and not being able to go to social events. Individuals with higher levels of education may have had increased levels of substance use due to more isolation.

While a limitation of this study is the lack of longitudinal HIV outcomes data, strengths include the large sample size and the use of laboratory results from the DC Cohort database. Future directions include examining the impact of psychosocial factors on the entire HIV care cascade and generating evidence for measures supporting mental health and easing the psychosocial impact of the pandemic among PWH [16, 54, 55]. In conclusion, we must ensure that the most vulnerable have access to the resources they need to maintain their health as the pandemic persists.

## Supplementary Information

**Additional file 1: Appendix 1.**

## Data Availability

The datasets generated and/or analysed during the current study are not publicly available to protect the confidentiality of participants but are available from the corresponding author on reasonable request.
