# Supplementary material for: Psychosocial impacts of the COVID-19 pandemic from a cross-sectional Survey of people living with HIV in Washington, DC
Source: AIDS Res Ther. 2023 May 9;20:27. doi: 10.1186/s12981-023-00517-z (PMC10169119; doi:10.1186/s12981-023-00517-z)
Supplement: Supplementary file 1 — Additional file 1: Appendix 1. [file 12981_2023_517_MOESM1_ESM.docx]

# Additional file 1: Appendix 1.

***Survey***

Do you agree to participate in this study?

Yes

No

Today's date

In the next field, please enter the unique ID you received via email or text along with the survey link. The ID begins with the letters 'CO' and must be entered correctly so we know who to pay.

Participant ID

At which site were you notified about this study?

Children’s National Medical Center Pediatric Clinic

Children's National Medical Center Adolescent Clinic

Family and Medical Counseling Service

Georgetown University

George Washington Medical Faculty Associates

Howard University Hospital Adult Clinic

Howard University Hospital Pediatric Clinic

Kaiser Permanente

La Clinica Del Pueblo

MetroHealth

Washington Health Institute

Washington Hospital Center

Whitman-Walker Health

Unity Health Care

Veterans Affairs Medical Center

**SECTION A. RACE, GENDER**

**In this section we will ask you about gender and race, education, employment, and living arrangements. All answers will remain confidential.**

How old are you today?

Do you identify as:

Female

Male

Intersex

Transgender female (male-to-female)

Transgender man (female-to-male)

Genderqueer/ Gender nonconforming (neither exclusively male or female)

Other

Decline to answer

If other, please specify:

Are you Hispanic, Latino, or of Spanish origin?

Yes

No

Decline to answer

Which of these groups would you say best represents your race? Please select all that apply.

American Indian or Alaska Native Asian

Black or African American

Native Hawaiian or other Pacific Islander

White or Caucasian

Bi/multiracial

Other

Don't know/Not sure

Decline to answer

If other, please specify:

What is the highest grade or year of school you completed?

Less than a high school diploma

Grade 12 or GED (High school graduate)

College 1 year to 3 years (Some college or technical school)

College 4 years or more (College graduate)

Decline to answer

Choose the answer that best applies to your current relationship status. Are you:

Divorced or marriage annulled

Legally or Common-law married

Not Married, but living with a partner

Separated

Single, Never married Widowed

Other

Decline to answer

If other, please specify:

Where are you living now?

In a rented house or apartment

In your own home or condominium

At your parent's house

Someone else's house or apartment

In a rooming, boarding, or group home

In a shelter or welfare hotel

On the street(s)

In a residential drug, alcohol treatment facility

In an assisted living or senior living community

In an unstable or temporary housing situation

Other place

If other, please specify:

In what state do you currently live?

District of Columbia

Maryland

Virginia

West Virginia

Other

Decline to answer

If other, please specify:

If you live in the District of Columbia, in which ward do you live?

1

2

3

4

5

6

7

8

Don't know/not sure

Decline to answer

What is the zip code of your home address?

(Enter 00000 if unknown)

How many bedrooms are in your home?

0/studio/efficiency apartment

1

2

3

4 or more

How many people currently live in your household?

(Including yourself, spouse/partner, children, and others who live in your home)?

1 (live alone)

2

3

4

5

6

7

8

9 or more

How many members of your household, including yourself, are between the ages of 18-29 years of age?

How many members of your household, including yourself, are between the ages of 30-64 years of age?

How many members of your household, including yourself, are between the ages of 65-79 years of age?

How many members of your household, including yourself, are aged 80 or older?

**SECTION B. MEDICAL HISTORY**

**The following questions ask about health conditions you may have. All answers will remain confidential.**

Have you received any HIV care since March 1, 2020?

Yes

No

To your knowledge, have you ever had any of the following medical conditions? Please select all that apply.

Angina or coronary heart disease

Asthma

Cancer

Chronic lung disease including C.O.P.D., emphysema or chronic bronchitis

Depression

Heart attack also called a myocardial infarction

High blood pressure

Kidney disease (not including kidney stones, bladder infection or incontinence)

Overweight or obesity

Stroke

Type 2 diabetes

I have not been told that I have any of the above conditions

Decline to answer

To your knowledge, are you now pregnant?

Yes

No

Don't know / Not sure

Decline to answer

During the past 12 months, have you had either a flu vaccine that was sprayed in your nose or a flu shot injected into your arm?

Yes

No

Don't know / Not sure

Decline to answer

**Please indicate how frequently you smoke the following: (**Never smoked, Previously smoked, Currently smoke)

Cigarettes E-cigarettes

Vape pens, personal vaporizers

Marijuana

**SECTION C. COVID-19 SYMPTOMS AND TESTING**

**The following questions will ask you about your symptoms and testing related to the novel coronavirus (COVID-19). All answers will remain confidential.**

Since March 1, 2020 do you think you have had symptoms of COVID-19?

Yes

No

Have you had any of the following symptoms since March 1, 2020? Please select all that apply.

Blistering of toes/feet

Chest pain

Chills

Confusion
Cough

Diarrhea

Fatigue or excessive sleepiness

Fever

Headache

Loss of smell

Loss of taste

Muscle or body aches

Nausea

Rash

Runny nose

Shortness of breath

Sore throat

Vomiting

Other

No symptoms

If other, please specify:

What was the date of your first symptom?

(Select the 15th day of the month if unknown)

Have you had a test for COVID-19 (including nasal, oral or blood tests, but not antibody tests)?

Yes

No

Were you tested by your HIV primary care doctor?

Yes

No

Was the test result positive?

Yes, the test was positive

No, the test was negative

Still waiting for results

What was the date of the test?

(Select the 15th day of the month if unknown)

Where did you get tested?

COVID testing tent

Drive-through testing

Emergency Department

Hospital, while inpatient

Someone came to my house to test me

Urgent care

Other

Decline to answer

If other, please specify:

Were you ever hospitalized since March 1, 2020 because of your COVID-19 symptoms?

Yes

No

Decline to answer

Where were you hospitalized?

Children's National Medical Center

George Washington University Hospital

Holy Cross Hospital

Howard University Hospital

Medstar Georgetown University Hospital

Medstar Washington Hospital Center

PG County Hospital Sibley Hospital

United Medical Center

Veterans Affairs Medical Center

Virginia Hospital Center

Other

Decline to answer

If other, please specify:

When you were in quarantine or isolation for COVID-19, did you have a separate room for sleeping and daily activities where you can stay away from others in your household?

Yes

No

Decline to answer

Have you received a COVID-19 vaccine?

[If yes] Which vaccine did you receive?

Pfizer

Moderna

AstraZeneca

Johnson and Johnson

Other

If other, please specify:

[If yes] How many doses have you received?

One

Two

[If yes] Where were you vaccinated?

HIV provider

Primary care provider

Hospital (e.g., United Medical Center, Medstar Washington Hospital Center)

Pharmacy (e.g., Giant, Safeway, Walmart)

Community health center (e.g. Unity, Whitman-Walker Health)

Local health department

Church or faith-based organization (e.g., Pennsylvania Avenue Baptist Church)

Mass vaccination site (e.g. sports arena or stadium, amusement park)

Other

If other, please specify:

[If yes] Did you experience any of the following symptoms after you were vaccinated?

pain at the injection site

fever

chills

tiredness/fatigue

headache

muscle pain

joint pain

swollen lymph nodes in the same arm as the injection

nausea

vomiting

I had no symptoms after vaccination.

Bell’s palsy

Anaphylaxis

Other

If other, please specify

[If no] Would you get a vaccine for COVID-19 if it was available today?

Yes

No

Don't know / Not sure

Decline to answer

[If no, don’t know] For each response below, please indicate if it is a major or minor reason why you do not want to get a vaccine to prevent COVID-19. (Major, Minor, not a reason)

Concern about side effects

Concern that the vaccine is being developed too quickly

The vaccine could give me COVID

Want to know more about how well it works

Plan to wait and see if it is safe and may get it later

Don’t trust the government

Plan to use masks/other precautions instead

Don’t think it will protect you against new variants

Don't think you need it

Had COVID-19 and should be immune

It would cost too much

Doctor has not recommended a COVID-19 vaccine to me

**SECTION D. COVID-19 SYMPTOMS AND TESTING AMONG HOUSEHOLD CONTACTS**

**The following questions will ask you about symptoms and testing related to**

**COVID-19/coronavirus among people living in your household. All answers will remain confidential.**

Not including yourself, how many members of your household have had symptoms of COVID-19 (for example, a fever, cough, or shortness of breath, chills, sore throat, loss of taste or smell) since March 1, 2020?

N/A - I live alone

None1

2

3

4

5

6

7

8

9 or more

Not including yourself, how many members of your household have received a nasal, oral swab, or blood test for COVID-19 since March1st, 2020?

None

1

2

3

4

5

6

7

8

9 or more

Decline to answer

Not including yourself, how many members of your household have had a positive test result since March 1, 2020?

None

1

2

3

4

5

6

7

8

9 or more

Decline to answer

Since March 1, 2020, have you been in close contact with a person outside of your household who has tested positive for COVID-19? A close contact is defined as someone you were within 6 feet of for at least 15 minutes.

Yes

No

Don't know/Not sure

Decline to answer

**SECTION E. IMPACT OF COVID-19**

**The following questions will ask you the impact that the novel coronavirus (COVID-19) has had on you. All answers will remain confidential.**

What was your work status on January 1, 2020? Please select all that apply.

Disabled

Homemaker

Full-time employed

Full-time student

Part-time employed

Part-time student

Retired

Unemployed

Other

Decline to answer

If other, please specify:

If you are still employed full or part-time, have you been able to telework (i.e., work from my home or remotely) at least a few days per week?

Yes

No

Decline to answer

If you are still employed full or part-time, do you work in any of these essential service areas? Please select all that apply.

Construction

Delivery service (FedEx, UPS, etc.)

Food delivery

Grocery store

Healthcare (i.e., medical facility, pharmacy)

Hospitality (i.e., hotels, restaurants, casinos, etc.)

Mail carrier

Manufacturing (i.e., warehouse, factory)

Transportation (e.g., public, MTA, WMATA, RideOn, ride share)

Other

Do not work in an essential service area

Decline to answer

If other, please specify:

What was your annual household income from all sources on January 1, 2020?

How many people did this income support?

One, only myself

Two

Three

Four or more

Decline to answer

Since March 1, 2020 has your household income decreased as a result of COVID-19?

Yes

No

Which of the following contributed to a personal loss of income? Please select all that apply.

Business temporarily closed

I could not work and care for a child in the household

I felt I was at high risk and did not want to leave the home

I was fired/laid off

I was given time off with reduced pay (employer provided benefits)

I was given time off without pay (not fired, but not working)

My hours were reduced

Other

Decline to answer

If other, please specify:

Have you lost your health insurance at any point since March 1, 2020?

Yes

No

Decline to answer

Have you lost your housing at any point since March 1, 2020?

Yes

No

Decline to answer

Since March 1, 2020, do you feel that your ability to access non-HIV related care has been negatively impacted?

Yes

No

Don't know / Not sure

Decline to answer

Which of the following non-HIV related services have you been unable to access since March 1, 2020? Please select all that apply.

Addiction services

Aging services (senior center/adult day care)

Dentist

Disability services

Elective surgery

Home health care

Non-HIV related medications

Non-HIV specialty care (e.g., dermatology, orthopedics,)

Mental health care

Primary care

Reproductive health care

Social services (e.g., case management)

Other

If other, please specify:

**To what degree has COVID-19 and the plans used to manage COVID-19 increased or decreased your ability to access the following HIV-related services? (**Decreased, No change, Increased**)**

Making HIV care appointments.

Keeping HIV care appointments.

Getting viral loads or other labs done.

Getting HIV medication prescriptions filled/mailed to me.

Daily adherence to HIV meds.

Accessing STI testing or prevention (e.g., condoms).

Since March 1, 2020 have any of the following been negatively impacted by COVID-19? Please select all that apply.

Paying rent/mortgage.

Getting the food you need.

Getting to a pharmacy.

Getting to a clinic or doctor's appointment.

Going to work or attending school.

Using public transportation.

Accessing a stable internet connection.

Supporting other family/partners who have lost jobs.

**To what degree has COVID-19 and the plans used to manage COVID-19 increased or decreased the following? (**Decreased, No change, Increased, Decline to answer**)**

Number of sexual partners. Opportunities to have sex.

Your use of dating/hook-up apps to connect virtually with other people.

Access to condoms. Access to contraception. Use of condoms.

Your use of dating/hook-up apps to meet other people in person.

Access to STI testing or treatment.

Use of recreational drugs. Alcohol consumption.

Tell us how else COVID-19 is impacting your life:

**SECTION F. RISK PERCEPTION**

**The following questions will ask you about your perceived risk of contracting COVID-19/coronavirus. All answers will remain confidential.**

How worried are you about getting sick from the novel coronavirus (COVID-19)? Would you say:

Not at all worried

Not too worried

Somewhat worried

Very worried

Decline to answer

How worried are you about your loved ones getting sick from the novel coronavirus (COVID-19)? Would you say:

Not at all worried

Not too worried

Somewhat worried

Very worried

Decline to answer

Do you personally know anyone who has died from COVID-19?

Yes

No

Don't know / Not sure

Decline to answer

**SECTION G. STIGMA**

**The following questions will ask you about any disapproval or discrimination that you may have experienced as a result of being diagnosed with COVID-19. All answers will remain confidential.**

**For the following statements, please indicate whether you agree or disagree:**

**(**Agree, Disagree, Decline to answer**)**

It is/was difficult to tell people about my COVID-19 diagnosis.

Being diagnosed with COVID-19 makes/made me feel dirty.

I feel/felt guilty that I have COVID-19

I am/was ashamed that I have COVID-19.

I sometimes feel/felt worthless because I have/had COVID-19.

I hide/hid my COVID-19 diagnosis from others.

In the time immediately after being diagnosed with COVID-19, please indicate if you were made to feel less than others because of any of the following.

Please select all that apply.

Gender

Poverty

Homelessness

Race/ethnicity

Immigration status

Sexual Preference

Decline to answer

**SECTION H. ANXIETY AND DEPRESSION**

**The following questions will ask you about any symptoms of anxiety and depression that you may have experienced. All answers will remain confidential.**

**(**Highly decreased, Somewhat decreased, No change, Somewhat increased, Highly increased)

General quality of life.

Feeling anxious.

Quality of sleep (e.g., not being able to sleep well)

Feeling connected to family.

Feeling connected to friends.

**How often since March 1, 2020 have you...**

**(**Not at all, Several days, More than half the days, Nearly every day**)**

Had little interest or pleasure in doing things.

Felt down, depressed or hopeless.

**SECTION I. TELEHEALTH**

**The following questions will ask you about any HIV care visits that you may have had virtually. All answers will remain confidential.**

Did you engage with your HIV provider using telehealth? (A telehealth or virtual visit means talking with an HIV care provider by phone or over the internet using a computer or smartphone instead of actually being in their office.)

Yes

No

What was your primary motivation for using telehealth?

Curiosity

Easier follow-up care

Faster, more convenient and comfortable

Had an urgent healthcare matter that could not wait for an in-person visit

I was told to quarantine and could not leave the house

Lower cost

More collaboration of health care professionals

Offered, recommended, or required by my healthcare provider

Reduced risk of exposure to COVID-19

Satisfied with previous telehealth visit

Other

Decline to answer

If other, please specify

How did you engage with your provider? Please select all that apply,

Video call on my computer

Video call on my phone

Using an app on my computer

Using an app on my phone

Telephone call

Other

If other, please specify:

How many visits have you had with your provider using a video call on your computer?

How many visits have you had with your provider using a video call on your phone?

How many visits have you had with your provider using an app on your computer?

How many visits have you had with your provider using an app on your phone?

How many visits have you had with your provider via a telephone call?

How many visits have you had with your provider in some other way?

How many providers were on your last telehealth visit?

One

Two

Three

Four +

Decline to answer

**For each item, please indicate how strongly you agree or disagree:**

**(**Disagree, Neither agree or disagree, Agree, N/A**)**

The telehealth visit was as good as a traditional in-person visit.

Using the telehealth service was convenient.

Being on camera made me feel embarrassed or uncomfortable.

I was worried about my privacy being on camera, in front of other people in my household and whether it was being recorded.

I was overall satisfied with my telehealth experience.

I will use telehealth in the future.

Please discuss any additional downsides or concerns with telehealth.

Please discuss any additional advantages of telehealth.

Please provide any additional comments or suggestions about your experience with telehealth.

Would you be willing to allow us to contact you in the future for additional COVID-19 related surveys or studies?

Yes

No

Please select the type of gift card you prefer:

An electronic gift card (emailed to you)

A physical gift card (mailed to your home address)

1 COVID-19 Map. Johns Hopkins Coronavirus Resour. Cent. https://coronavirus.jhu.edu/map.html (accessed 3 Jun 2022).

2 Wang C, Pan R, Wan X, Tan Y, Xu L, Ho CS, *et al.* **Immediate Psychological Responses and Associated Factors during the Initial Stage of the 2019 Coronavirus Disease (COVID-19) Epidemic among the General Population in China.** *Int J Environ Res Public Health* 2020; **17**. doi:10.3390/ijerph17051729

3 Qiu J, Shen B, Zhao M, Wang Z, Xie B, Xu Y. **A nationwide survey of psychological distress among Chinese people in the COVID-19 epidemic: implications and policy recommendations.** *Gen Psychiatry* 2020; **33**:e100213.

4 Shigemura J, Ursano RJ, Morganstein JC, Kurosawa M, Benedek DM. **Public responses to the novel 2019 coronavirus (2019-nCoV) in Japan: Mental health consequences and target populations.** *Psychiatry Clin Neurosci* 2020; **74**:281–282.

5 Li W, Yang Y, Liu Z-H, Zhao Y-J, Zhang Q, Zhang L, *et al.* **Progression of Mental Health Services during the COVID-19 Outbreak in China.** *Int J Biol Sci* 2020; **16**:1732–1738.

6 Jiloha R. **COVID-19 and mental health**. *Epidemiol Int E-ISSN 2455-7048* 2020; **5**:7–9.

7 WHO. **No Title**.

8 CDC. COVID-19 and Your Health. Cent. Dis. Control Prev. 2022.https://www.cdc.gov/coronavirus/2019-ncov/prevent-getting-sick/prevention.html (accessed 3 Jun2022).

9 Brooks SK, Webster RK, Smith LE, Woodland L, Wessely S, Greenberg N, *et al.* **The psychological impact of quarantine and how to reduce it: rapid review of the evidence.** *Lancet Lond Engl* 2020; **395**:912–920.

10 Abel T, McQueen D. The COVID-19 pandemic calls for spatial distancing and social closeness: not for social distancing! Int. J. Public Health. 2020; **65**:231–231.

11 Keeping your distance to stay safe. https://www.apa.org. https://www.apa.org/practice/programs/dmhi/research-information/social-distancing (accessed 3 Jun2022).

12 Orlando M, Burnam MA, Beckman R, Morton SC, London AS, Bing EG, *et al.* **Re-estimating the prevalence of psychiatric disorders in a nationally representative sample of persons receiving care for HIV: results from the HIV Cost and Services Utilization Study.** *Int J Methods Psychiatr Res* 2002; **11**:75–82.

13 Weaver MR, Conover CJ, Proescholdbell RJ, Arno PS, Ang A, Ettner SL. **Utilization of mental health and substance abuse care for people living with HIV/AIDS, chronic mental illness, and substance abuse disorders**. *JAIDS J Acquir Immune Defic Syndr* 2008; **47**:449–458.

14 Winwood JJ, Fitzgerald L, Gardiner B, Hannan K, Howard C, Mutch A. **Exploring the Social Impacts of the COVID-19 Pandemic on People Living with HIV (PLHIV): A Scoping Review.** *AIDS Behav* 2021; **25**:4125–4140.

15 Barbera LK, Kamis KF, Rowan SE, Davis AJ, Shehata S, Carlson JJ, *et al.* **HIV and COVID-19: review of clinical course and outcomes.** *HIV Res Clin Pract* 2021; **22**:102–118.

16 Sherbuk JE, Williams B, McManus KA, Dillingham R. **Financial, food, and housing insecurity due to coronavirus disease 2019 among at-risk people with human immunodeficiency virus in a nonurban Ryan white HIV/AIDS program clinic**. *Open Forum Infect Dis* 2020; **7**:1–5.

17 Jones DL, Ballivian J, Rodriguez VJ, Uribe C, Cecchini D, Salazar AS, *et al.* **Mental health, coping, and social support among people living with HIV in the Americas: a comparative study between Argentina and the USA during the SARS-CoV-2 pandemic**. *AIDS Behav* 2021; **25**:2391–2399.

18 Ballivian J, Alcaide ML, Cecchini D, Jones DL, Abbamonte JM, Cassetti I. **Impact of COVID-19-Related Stress and Lockdown on Mental Health Among People Living With HIV in Argentina.** *J Acquir Immune Defic Syndr 1999* 2020; **85**:475–482.

19 Hochstatter KR, Akhtar WZ, Dietz S, Pe-Romashko K, Gustafson DH, Shah DV, *et al.* **Potential Influences of the COVID-19 Pandemic on Drug Use and HIV Care Among People Living with HIV and Substance Use Disorders: Experience from a Pilot mHealth Intervention.** *AIDS Behav* 2021; **25**:354–359.

20 Marziali ME, Card KG, McLinden T, Wang L, Trigg J, Hogg RS. **Physical Distancing in COVID-19 May Exacerbate Experiences of Social Isolation among People Living with HIV.** *AIDS Behav* 2020; **24**:2250–2252.

21 Jones DL, Morgan KE, Martinez PC, Rodriguez VJ, Vazquez A, Raccamarich PD, *et al.* **COVID-19 Burden and Risk Among People With HIV.** *J Acquir Immune Defic Syndr 1999* 2021; **87**:869–874.

22 Siewe Fodjo JN, Faria de Moura Villela E, Van Hees S, Vanholder P, Reyntiens P, Colebunders R. **Follow-Up Survey of the Impact of COVID-19 on People Living with HIV during the Second Semester of the Pandemic.** *Int J Environ Res Public Health* 2021; **18**. doi:10.3390/ijerph18094635

23 Cooley SA, Nelson B, Doyle J, Rosenow A, Ances BM. **Collateral damage: Impact of SARS-CoV-2 pandemic in people living with HIV.** *J Neurovirol* 2021; **27**:168–170.

24 Wion RK, Miller WR. **The Impact of COVID-19 on HIV Self-Management, Affective Symptoms, and Stress in People Living with HIV in the United States.** *AIDS Behav* 2021; **25**:3034–3044.

25 Deaths in the DMV increase as officials warn it could be next hot spot. https://www.washingtonpost.com/local/deaths-in-dmv-increase-as-officials-warn-it-could-be-next-hot-spot/2020/04/06/9863defa-77b2-11ea-b6ff-597f170df8f8_story.html

26 Officials Warn D.C. Could Be The Next Coronavirus ‘Hot Spot.’ What Does That Mean? https://dcist.com/story/20/04/07/officials-warn-d-c-could-be-the-next-coronavirus-hot-spot-what-does-that-mean/

27 White House says D.C. region among worst in country, as summer closures continue. https://www.washingtonpost.com/local/white-house-says-dc-region-among-worst-in-country-as-summer-closures-continue/2020/05/22/31e4cc8c-9c3a-11ea-ac72-3841fcc9b35f_story.html

28 HHS. Ending the HIV Epidemic Counties and Territories. 2019.

29 Kaufman MR, Cornish F, Zimmerman RS, Johnson BT. **Health Behavior Change Models for HIV Prevention and AIDS Care: Practical Recommendations for a Multi-Level Approach**. *JAIDS J Acquir Immune Defic Syndr* 2014; **66**:S250.

30. Kaiser Permanente Research Bank COVID-19 Surveys. 2020; https://researchbank.kaiserpermanente.org/wp-content/uploads/2021/05/KPRB-COVID-19-Survey_-summary.pdf

31 Wilbourn B, Saafir-Callaway B, Jair K, Wertheim JO, Laeyendeker O, Jordan JA, et al. Characterization of HIV Risk Behaviors and Clusters Using HIV-Transmission Cluster Engine Among a Cohort of Persons Living with HIV in Washington, DC. AIDS Res Hum Retroviruses 2021; 37:706–715.

32 Stanford University. COVID19 Interview Items for Vulnerable Populations. 2020; Available from https://clelandcm.github.io/COVID19-Interview-Items/COVID-Items.html#stanford (accessed 3 Jun2022).

33 Kalichman S. Kalichman Covid-19 Assessment. 2020; Available from https://clelandcm.github.io/COVID19-Interview-Items/COVID-Items.html#kalichman-covid-19-assessment (accessed 3 Jun2022).

34 COVID-19 Household Environment Scale. Available from https://elcentro.sonhs.miami.edu/research/measures-library/ches/ches-eng/index.html (accessed 3 Jun2022).

35 ATN COVID Questionnaire. https://clelandcm.github.io/COVID19-Interview-Items/COVID-Items.html#atn

36 Human Infection with 2019 Novel Coronavirus Case Report Form Interviewer Information Case Classification and Identification. https://www.cdc.gov/coronavirus/2019-ncov/downloads/pui-form.pdf

37 Baseline Questionnaire for the Communities, Households and SARS/COV-2 Epidemiology (CHASE) COVID Study. 2020. https://cunyisph.org/wp-content/uploads/CHASE-COVID_baseline_V2.1.pdf

38 Kroenke K, Spitzer RL, Williams JBW. **The Patient Health Questionnaire-2: validity of a two-item depression screener.** *Med Care* 2003; **41**:1284–1292.

39 District of Columbia Eligible Metropolitan Area Integrated HIV/AIDS prevention and care plan. https://doh.dc.gov/sites/default/files/dc/sites/doh/service_content/attachments/DC%20DOH%20INTEGRATED%20PLAN_FINAL.pdf.pdf

40 Wainwright JJ, Beer L, Tie Y, Fagan JL, Dean HD. **Socioeconomic, Behavioral, and Clinical Characteristics of Persons Living with HIV Who Experience Homelessness in the United States, 2015-2016.** *AIDS Behav* 2020; **24**:1701–1708.

41 Aidala AA, Lee G, Abramson DM, Messeri P, Siegler A. **Housing need, housing assistance, and connection to HIV medical care**. *AIDS Behav* 2007; **11**:101–115.

42 Kidder DP, Wolitski RJ, Campsmith ML, Nakamura GV. **Health status, health care use, medication use, and medication adherence among homeless and housed people living with HIV/AIDS**. *Am J Public Health* 2007; **97**:2238–2245.

43 Kunzler AM, Röthke N, Günthner L, Stoffers-Winterling J, Tüscher O, Coenen M, *et al.* **Mental burden and its risk and protective factors during the early phase of the SARS-CoV-2 pandemic: systematic review and meta-analyses.** *Glob Health* 2021; **17**:34.

44 Sanchez TH, Zlotorzynska M, Rai M, Baral SD. **Characterizing the Impact of COVID-19 on Men Who Have Sex with Men Across the United States in April, 2020.** *AIDS Behav* 2020; **24**:2024–2032.

45 Schaaf REA, Verburgh ML, Boyd A, Wit FW, Nieuwkerk PT, Schim van der Loeff MF, *et al.* **Change in substance use and the effects of social distancing on health-related quality of life and depressive symptoms during the COVID-19 pandemic in people living with and without HIV.** *JAIDS J Acquir Immune Defic Syndr* Published Online First: 9900.https://journals.lww.com/jaids/Fulltext/9900/Change_in_substance_use_and_the_effects_of_social.77.aspx

46 Grov C, Golub SA, Parsons JT, Brennan M, Karpiak SE. **Loneliness and HIV-related stigma explain depression among older HIV-positive adults**. *AIDS Care* 2010; **22**:630–639.

47 Czeisler MÉ. **Mental Health, Substance Use, and Suicidal Ideation During the COVID-19 Pandemic — United States, June 24–30, 2020**. *MMWR Morb Mortal Wkly Rep* 2020; **69**. doi:10.15585/mmwr.mm6932a1

48 Ups and Downs of Daily Life During COVID-19: Age Differences in Affect, Stress, and Positive Events | The Journals of Gerontology: Series B | Oxford Academic. https://academic.oup.com/psychsocgerontology/article/76/2/e30/5872612?login=false (accessed 18 Aug2022).

49 van Tilburg TG, Steinmetz S, Stolte E, van der Roest H, de Vries DH. **Loneliness and Mental Health During the COVID-19 Pandemic: A Study Among Dutch Older Adults**. *J Gerontol Ser B* 2021; **76**:e249–e255.

50 Vahia IV, Jeste DV, Reynolds CF III. **Older Adults and the Mental Health Effects of COVID-19**. *JAMA* 2020; **324**:2253–2254.

51 Pearman A, Hughes ML, Smith EL, Neupert SD. **Age Differences in Risk and Resilience Factors in COVID-19-Related Stress**. *J Gerontol Ser B* 2021; **76**:e38–e44.

52 Kapogiannis BG, Koenig LJ, Xu J, Mayer KH, Loeb J, Greenberg L, *et al.* **The HIV Continuum of Care for Adolescents and Young Adults Attending 13 Urban US HIV Care Centers of the NICHD-ATN-CDC-HRSA SMILE Collaborative**. *JAIDS J Acquir Immune Defic Syndr* 2020; **84**:92–100.

53 COVID-19 recovery in hardest-hit sectors could take more than 5 years | McKinsey & Company. https://www.mckinsey.com/featured-insights/coronavirus-leading-through-the-crisis/charting-the-path-to-the-next-normal/covid-19-recovery-in-hardest-hit-sectors-could-take-more-than-5-years (accessed 18 Aug2022).

54 Davison KM, Thakkar V, Lin SL, Stabler L, MacPhee M, Carroll S, *et al.* **Interventions to Support Mental Health among Those with Health Conditions That Present Risk for Severe Infection from Coronavirus Disease 2019 (COVID-19): A Scoping Review of English and Chinese-Language Literature.** *Int J Environ Res Public Health* 2021; **18**. doi:10.3390/ijerph18147265

55 Armbruster M, Fields EL, Campbell N, Griffith DC, Kouoh AM, Knott-Grasso MA, *et al.* **Addressing Health Inequities Exacerbated by COVID-19 Among Youth With HIV: Expanding Our Toolkit.** *J Adolesc Health Off Publ Soc Adolesc Med* 2020; **67**:290–295.
